# Sigmoid colon perforation caused by ingested removable dentures: A case report and literature review

**DOI:** 10.1097/MD.0000000000047429

**Published:** 2026-01-30

**Authors:** Junyan Fang, Chen Xu, Yuzhen Wen, Qiang Shao, Xiaohui Song

**Affiliations:** a Department of Stomatology, The Ninth People’s Hospital of Zibo and People’s Hospital of Huantai County, Zibo, P.R. China; b Department of Oral and Maxillofacial Surgery, Zibo Central Hospital, Zibo, China; c Department of Colorectal Surgery, The Ninth People’s Hospital of Zibo and People’s Hospital of Huantai County, Zibo, P.R. China.

**Keywords:** bowel perforation, foreign body ingestion, Hartmann procedure, laparoscopy, removable dentures

## Abstract

**Rationale:**

Ingestion of removable dentures leading to gastrointestinal perforation is a rare but potentially life-threatening emergency, particularly in the elderly population due to diminished oral sensation and swallowing reflex. This article reports the management of a patient who developed sigmoid colon necrosis and perforation.

**Patient concerns:**

A 76-year-old female presented to the emergency department with a 2-day history of nausea and left lower abdominal pain. Medical history collection confirmed that the patient had accidentally swallowed her removable denture 3 days ago.

**Diagnoses:**

Abdominal computed tomography identified a high-density foreign body in the sigmoid colon and pneumoperitoneum with dilated bowel loops. Combined with the medical history, the diagnosis of sigmoid colon perforation caused by ingested dentures was confirmed.

**Interventions:**

Emergency treatment involved Hartmann procedure for resection of the necrotic bowel segment and colostomy. Four months later, laparoscopic-assisted stoma reversal was successfully performed to restore intestinal continuity.

**Outcomes:**

The patient recovered uneventfully and was discharged without complications. Hartmann procedure is an effective and safe treatment for colonic perforation that needs emergency treatment and cannot be anastomozed in 1 stage.

**Lessons:**

Early identification and individualized treatment are critical for patients with ingested dentures. Denture restoration for high-risk groups should be reconsidered or replaced with safer alternatives.

## 1. Introduction

Ingestion of foreign bodies is most commonly encountered in pediatric and elderly populations. Sharp objects, such as denture clasps, carry a perforation risk as high as 15% to 35%.^[1]^ Removable dentures, due to their large size and complex structure, are prone to impaction in narrow gastrointestinal segments, leading to intestinal wall ischemia, necrosis, and perforation. Despite advancements in endoscopic retrieval techniques, delayed presentation or cases complicated by perforation often necessitate surgical intervention. Currently, reports on staged surgical approaches (e.g., Hartmann procedure combined with laparoscopic stoma reversal) remain scarce. This is the 1st detailed report of Hartmann procedure combined with 2-stage laparoscopic closure in the treatment of sigmoid colon perforation caused by denture. We analyze the diagnosis and treatment process in detail and review the literature to provide reference for clinicians.

## 2. Case presentation

**Figure 1.**
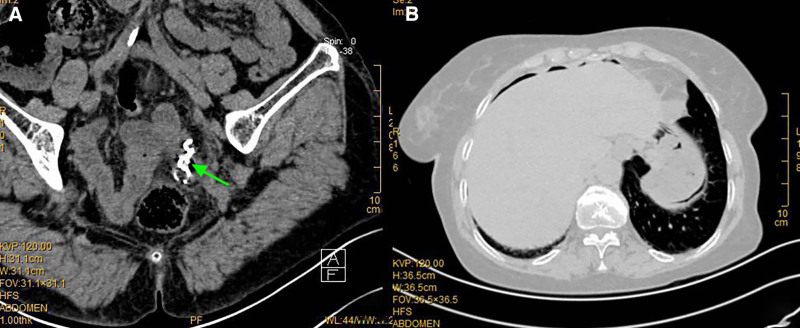
(A) CT showing a high-density foreign body (arrow) in the sigmoid colon. (B) Free air under the diaphragm and gas-distended bowel loops. CT = computed tomography.

A 76-year-old female presented to the emergency department on October 14, 2019, with a 2-day history of nausea and left lower abdominal pain. Physical examination revealed abdominal muscle guarding, tenderness, and rebound tenderness in the left lower quadrant. Laboratory tests showed leukocytosis (WBC 15.3 × 10⁹/L) and elevated C-reactive protein (98 mg/L). Abdominal computed tomography (CT) identified a high-density foreign body in the sigmoid colon (Fig. 1A) and pneumoperitoneum with dilated bowel loops (Fig. 1B). Further history confirmed accidental ingestion of a removable denture (single-tooth prosthesis with 2 metal tri-arm clasps) 3 days prior. Prompt surgical intervention was indicated.

Throughout the entire process of the case report, we have strictly adhered to the principle of patient confidentiality. Any identifiable details, such as the patient’s name in imaging materials, have not been presented.

### 2.1. First surgery (emergency exploratory laparotomy)

**Figure 2.**
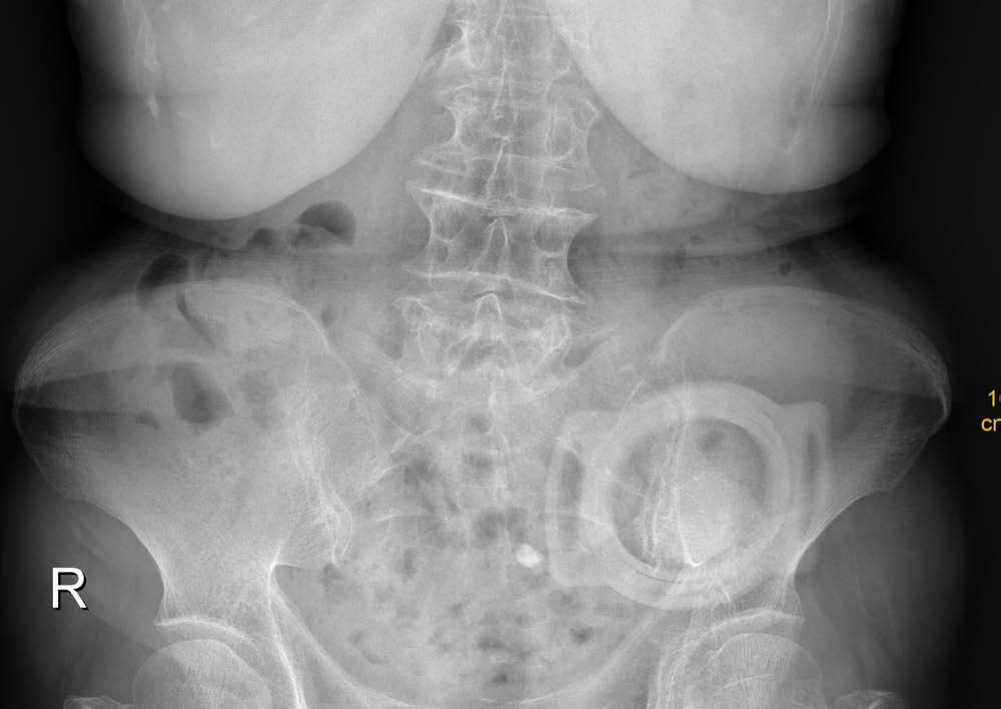
Abdominal radiograph showing no fluid levels or free air postoperatively.

Intraoperative findings included a 1.5-cm perforation in the mid-to-distal sigmoid colon with ischemic blackening of the bowel wall and purulent peritonitis. Resection of the necrotic segment (5 cm) was performed, followed by Hartmann procedure (distal closure and proximal colostomy). Histopathology confirmed full-thickness bowel wall necrosis with acute suppurative inflammation. Postoperative recovery was uneventful, with normalization of abdominal imaging (Fig. 2). The patient was discharged after 2 weeks.

### 2.2. Second surgery (laparoscopic-assisted stoma reversal)

**Figure 3.**
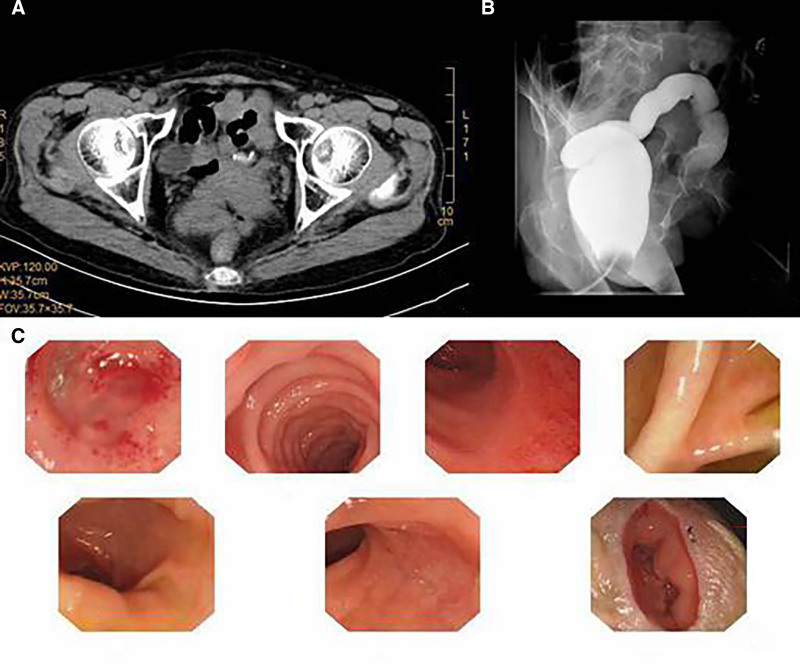
(A) CT demonstrating surgical clips at the colorectal anastomosis site. (B) Barium enema imaging demonstrated barium passage through the anastomotic fistula into the sigmoid colon. The sigmoid colon appeared dilated, without evidence of stricture, obstruction, or filling defects. (C) Colonoscopy revealed normal mucosa from the descending colon to the ileocecal valve. Localized mild congestion and edema were observed in the mucosa of the rectum and residual sigmoid colon. A single exposed suture staple was visible at the left lower quadrant abdominal stoma. CT = computed tomography.

**Figure 4.**
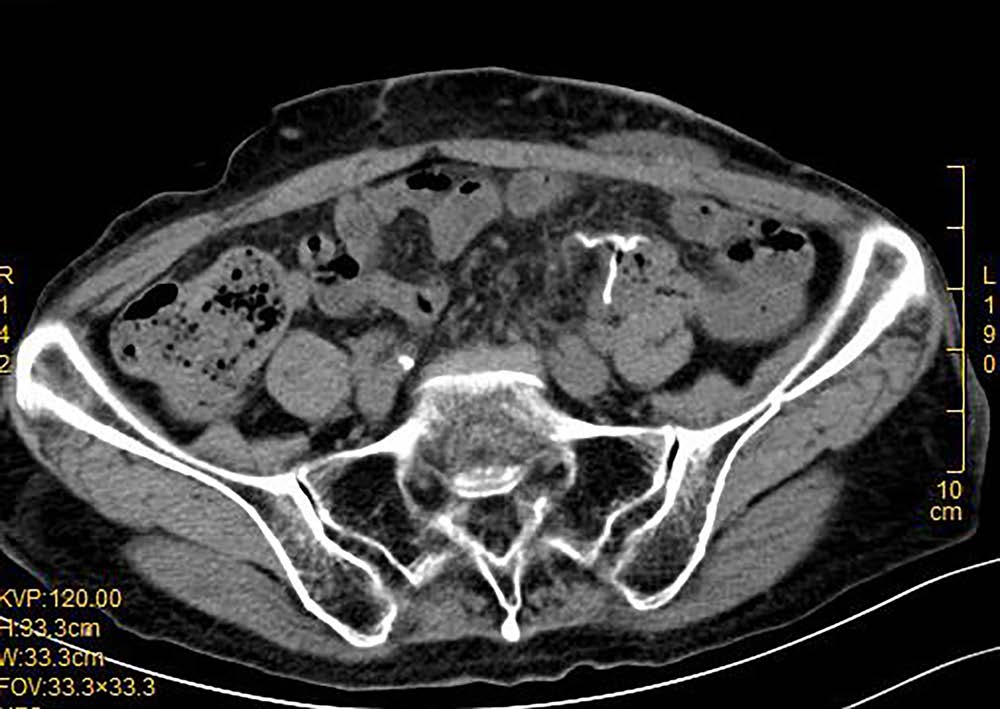
Two weeks postoperatively, follow-up CT scan demonstrated normal positioning of the anastomotic staples in the sigmoid colon, with well-defined surrounding fat planes. CT = computed tomography.

Four months later (February 24, 2020), the patient underwent laparoscopic adhesiolysis and colorectal anastomosis between the descending colon and residual sigmoid colon. Preoperative evaluation included CT (Fig. 3A), barium enema (Fig. 3B), and colonoscopy (Fig. 3C), confirming readiness for reversal. Postoperative recovery was rapid, with flatus resumption on day 7. Postoperative follow-up CT scan at 2 weeks (Fig. 4) revealed no abnormalities. The patient was followed up for 6 months post-discharge, during which the digestive system functioned normally without any discomfort.

## 3. Discussion

### 3.1. Etiology and risk factors

Literature has identified denture use as the most common risk factor among affected populations.^[2]^ Other predisposing factors include pediatric age, edentulism, alcohol/drug abuse, dementia, visual impairment, rapid eating, dysphagia, maxillofacial trauma, and general anesthesia.^[3–5]^ Special attention should be paid to identifying high-risk groups (elderly people with reduced neuromuscular control, dementia, and psychological disorders).

Regarding foreign body types, patients who ingest sharp objects warrant special attention. Long, sharp objects (e.g., small bones and toothpicks) and metallic items (e.g., dentures with clasps) significantly increase the risk of perforation.^[6]^ A retrospective study of 56 cases involving ingestion of dentures with clasps reported that only 2 cases achieved spontaneous passage, 8 were removed endoscopically, while 23 developed perforation.^[7]^

Regarding the timing of medical consultation, studies indicate that foreign body retention exceeding 24 hours increases the risk of perforation threefold.^[6]^ Patients should immediately fast and seek medical attention promptly after accidental ingestion.

The patient, a 76-year-old female, exhibited multiple high-risk factors for foreign body ingestion attributable to age-related oral sensory impairment, decreased swallowing reflex, and cognitive decline. During long-term use of removable dentures, she failed to undergo regular examinations for denture stability. Afterwards, an examination by an oral specialist revealed that the denture was a removable partial denture for the upper 1st molar. The abutment teeth at the front and back were severely worn and had become loose. It was speculated that during use, a gap developed between the clasp and the abutment teeth, causing the clasp to loosen. Eventually, this led to the denture falling out during eating and being swallowed into the digestive tract. The denture contained 2 sharp metallic clasps, posing significant risk for mechanical injury to the intestinal wall. Delayed presentation 3 days post-ingestion led to intestinal wall ischemia, necrosis, and intra-abdominal infection.

### 3.2. Diagnostic and therapeutic strategies

Most patients, their family members, or caregivers can identify the occurrence of foreign body ingestion. Symptomatic individuals often present with gastrointestinal symptoms and signs, such as abdominal pain, distension, nausea, vomiting, localized tenderness in the abdomen, or signs of peritoneal irritation (e.g., abdominal muscle guarding and rebound tenderness) following perforation. It is important to note that infants, patients with dementia, or psychiatric disorders may exhibit nonspecific symptoms such as choking, refusal to eat, drooling, wheezing, blood-tinged saliva, or respiratory distress.^[8,9]^ In cases where a caregiver is unavailable, a clear diagnostic history may not be obtainable. We recommend prompt medical evaluation for any suspected or confirmed foreign body ingestion, regardless of symptom presence, to differentiate between ingestion and aspiration, and to facilitate timely assessment and management planning.

Most ingested foreign bodies (>80%) do not require intervention and pass spontaneously, while 10% to 20% necessitate non-surgical management (e.g., endoscopic removal). Only approximately 1% of cases require surgical intervention.^[10]^ Imaging diagnosis is indispensable, particularly CT and X-ray imaging, which are critical for detecting foreign bodies and associated complications. CT with 3D reconstruction offers exceptional sensitivity for localizing foreign bodies within the gastrointestinal tract and identifying complications.^[11]^ By combining CT data with patient-reported information about the ingested object (e.g., type, material), clinicians can characterize its morphology, length, and consistency to identify high-risk objects (e.g., sharp/pointed objects > 6 cm).^[12]^ X-ray imaging remains valuable for its convenience, speed, and utility in initial localization, monitoring during conservative treatment, and postoperative follow-up.

Sharp objects lodged in the esophagus constitute a medical emergency and should be removed within 24 hours, as delays reduce the likelihood of successful retrieval and increase risks of complications such as perforation.^[13]^ Once passing through the esophagus into the stomach, most foreign bodies (including sharp ones) typically pass uneventfully. However, sharp objects carry a 35% risk of perforation,^[14]^ necessitating early endoscopic removal if feasible from the stomach or proximal duodenum. Otherwise, daily X-ray monitoring is required to track the object’s progress, with surgical intervention considered for objects retained in the stomach for ≥1 week or in the intestines for ≥3 days.^[15]^ In the lower gastrointestinal tract, while endoscopic removal via colonoscopy has been reported,^[16]^ open surgery remains the primary approach due to inaccessible locations or complications like perforation and peritonitis.

Perforation and peritonitis are absolute indications for emergency surgery.^[12]^ In this case, the perforation site was located in the sigmoid colon, consistent with the commonly reported predilection sites for foreign body perforation.^[17]^ During the operation, it was found that the patient’s necrotic intestinal segment was as long as 5 cm. Due to the long defect and the infection, the condition did not meet the requirements for resection and primary anastomosis. Therefore, we adopted a staged surgical approach using the Hartmann procedure, which is primarily indicated for emergent management of colorectal lesions requiring resection without primary anastomosis. First-stage emergency intervention: laparotomy for foreign body removal, resection of necrotic bowel segments, creation of a proximal colostomy, and distal bowel closure. This strategy rapidly controlled infection, minimized operative time, and improved patient survival. Second-stage minimally invasive stoma reversal: 4 months later, laparoscopic-assisted descending colon-sigmoid colon anastomosis was performed to restore intestinal continuity, reduce postoperative trauma, and achieve favorable functional outcomes. We report in detail for the 1st time the staged surgical treatment of perforation of the sigmoid colon due to dentures.

### 3.3. Prevention recommendations

On the one hand, before carrying out denture restoration for patients, clinical physicians should conduct necessary evaluations of their cognitive and motor functions to identify high-risk groups (elderly people with reduced neuromuscular control, dementia, and psychological disorders). For patients with severe motor or psychological disorders, removable dentures should be prohibited, and safer alternatives (such as fixed dentures or implant dentures) should be adopted. On the other hand, denture design should avoid multi-clasp structures, instead adopting implant-supported dentures or monolithic denture designs. For elderly patients using removable dentures, it is crucial to emphasize avoiding sticky foods and conducting regular stability checks. In cases of accidental ingestion, immediate fasting and prompt imaging evaluation are essential to assess the situation and guide management.

### 3.4. Patient perspective

We asked the patient and his family members, and found that no family members were present when the elderly swallowed the denture by mistake. The elderly had a fluke mentality about the consequences after swallowing the denture, and did not inform his family members to help him seek medical advice in time. After this incident, the patient’s family members expressed that they should strengthen monitoring of the elderly, and take the elderly to the dental clinic for fixed or implant denture restoration if the elderly’s physical condition allowed.

## 4. Conclusion

Early identification and individualized treatment are critical for patients with ingested dentures. Staged surgical intervention following bowel perforation can balance emergency management with long-term functional recovery. Clinicians must conduct comprehensive evaluations to optimize preventive strategies and clinical workflows for high-risk populations. For dentists and prosthodontists, careful screening of patients before prescribing removable dentures is essential to prevent this life-threatening complication.

## Author contributions

**Conceptualization:** Junyan Fang, Xiaohui Song.

**Data curation:** Qiang Shao.

**Formal analysis:** Junyan Fang.

**Resources:** Yuzhen Wen.

**Validation:** Junyan Fang, Xiaohui Song.

**Writing – review & editing:** Junyan Fang, Xiaohui Song.

**Writing – original draft:** Chen Xu.

## References

[1] Hashmi S Walter J Smith W Latis S. Swallowed partial dentures. J R Soc Med. 2004;97:72–5. 14749401 10.1258/jrsm.97.2.72 PMC1079292

[2] Rodríguez-Hermosa JI Codina-Cazador A Sirvent JM Martín A Gironès J Garsot E. Surgically treated perforations of the gastrointestinal tract caused by ingested foreign bodies. Colorectal Dis. 2008;10:701–7. 18005196 10.1111/j.1463-1318.2007.01401.x

[3] Hodges ED Durham TM Stanley RT. Management of aspiration and swallowing incidents: a review of the literature and report of case. ASDC J Dent Child. 1992;59:413–9. 1491079

[4] Toshima T Morita M Sadanaga N. Surgical removal of a denture with sharp clasps impacted in the cervicothoracic esophagus: report of three cases. Surg Today. 2011;41:1275–9. 21874430 10.1007/s00595-010-4467-x

[5] Neustein S Beicke M. Ingestion of a fixed partial denture during general anesthesia. Anesth Prog. 2007;54:50–1. 17579503 10.2344/0003-3006(2007)54[50:IOAFPD]2.0.CO;2 PMC1893093

[6] Velitchkov NG Grigorov GI Losanoff JE Kjossev KT. Ingested foreign bodies of the gastrointestinal tract: retrospective analysis of 542 cases. World J Surg. 1996;20:1001–5. 8798356 10.1007/s002689900152

[7] Kajihara Y Kawaguchi A Ueda T. Case report: endoscopic removal of an ingested denture with clasp. Prog Dig Endosc. 2007;70:108–9.

[8] Hachimi-Idrissi S Corne L Vandenplas Y. Management of ingested foreign bodies in childhood: our experience and review of the literature. Eur J Emerg Med. 1998;5:319–23. 9827834

[9] Kamal I Thompson J Paquette DM. The hazards of vinyl glove ingestion in the mentally retarded patient with pica: new implications for surgical management. Can J Surg. 1999;42:201–4. 10372016 PMC3788950

[10] Abdullah M Cui J Hendaheva R. Sigmoid perforation caused by dentures – a rare case report. Int J Surg Case Rep. 2017;41:280–2. 29127915 10.1016/j.ijscr.2017.10.030 PMC5683739

[11] Takada M Kashiwagi R Sakane M Tabata F Kuroda Y. 3D-CT diagnosis for ingested foreign bodies. Am J Emerg Med. 2000;18:192–3. 10750930 10.1016/s0735-6757(00)90018-4

[12] Ikenberry SO Jue TL Anderson MA. Management of ingested foreign bodies and food impactions. Gastrointest Endosc. 2011;73:1085–91. 21628009 10.1016/j.gie.2010.11.010

[13] Park JH Park CH Park JH. Review of 209 cases of foreign bodies in the upper gastrointestinal tract and clinical factors for successful endoscopic removal. Korean J Gastroenterol. 2004;43:226–33. 15100486

[14] Ricote GC Torre LR De Ayala VP. Fiberendoscopic removal of foreign bodies of the upper part of the gastrointestinal tract. Surg Gynecol Obstet. 1985;160:499–504. 4002103

[15] Smith MT Wong RK. Foreign bodies. Gastrointest Endosc Clin N Am. 2007;17:361–82, vii. 17556153 10.1016/j.giec.2007.03.002

[16] Abe K Miki A Okamura T. Endoscopic removal of a denture with clasps impacted in the ileocecum. Clin J Gastroenterol. 2014;7:506–9. 25425499 10.1007/s12328-014-0539-6

[17] Flanagan M Clancy C Riordain MGO. Impaction of swallowed dentures in the sigmoid colon requiring sigmoid colectomy. Int J Surg Case Rep. 2018;47:89–91. 29753276 10.1016/j.ijscr.2018.04.033 PMC5994734

